# Memory, reasoning, and categorization: parallels and common mechanisms

**DOI:** 10.3389/fpsyg.2014.00529

**Published:** 2014-06-17

**Authors:** Brett K. Hayes, Evan Heit, Caren M. Rotello

**Affiliations:** ^1^School of Psychology, University of New South WalesSydney, NSW, Australia; ^2^School of Social Sciences, Humanities and Arts, University of CaliforniaMerced, CA, USA; ^3^Department of Psychology, University of MassachusettsAmherst, MA, USA

**Keywords:** reasoning, recognition memory, categorization, induction, deduction

## Abstract

Traditionally, memory, reasoning, and categorization have been treated as separate components of human cognition. We challenge this distinction, arguing that there is broad scope for crossover between the methods and theories developed for each task. The links between memory and reasoning are illustrated in a review of two lines of research. The first takes theoretical ideas (two-process accounts) and methodological tools (signal detection analysis, receiver operating characteristic curves) from memory research and applies them to important issues in reasoning research: relations between induction and deduction, and the belief bias effect. The second line of research introduces a task in which subjects make either memory or reasoning judgments for the same set of stimuli. Other than broader generalization for reasoning than memory, the results were similar for the two tasks, across a variety of experimental stimuli and manipulations. It was possible to simultaneously explain performance on both tasks within a single cognitive architecture, based on exemplar-based comparisons of similarity. The final sections explore evidence for empirical and processing links between inductive reasoning and categorization and between categorization and recognition. An important implication is that progress in all three of these fields will be expedited by further investigation of the many commonalities between these tasks.

## INTRODUCTION

There is little doubt that memory, categorization, and reasoning are all central components of human cognition. But traditionally they have been treated as *separate* components. Each has been studied using different paradigms and very different theoretical models have been developed to explain each activity. This separation extends to the way that students are taught about each topic; memory and reasoning, for example typically appear in different sections of *Cognitive Psychology* textbooks (Heit and Hayes, 2008).

To some extent this separation is warranted. Understanding the cognitive processes involved in any one of these areas represents a major scientific challenge. Much of our current understanding of human memory, reasoning, and categorization has only been achieved via the development of specialized methods and models in each domain. However, it could be argued that the fractionation of the study of human cognition sometimes obscures deeper commonalities between tasks and processes. Newell (1973) famously pointed out that the proliferation of phenomena studied in experimental psychology and the dichotomous division of psychological processes (e.g., continuous versus discrete representations, analog versus digital processing) can impede theory development and cumulative scientific progress. As a remedy Newell suggested that cognitive scientists should examine how a single processing system may be extended to explain a variety of disparate tasks [see Dale et al. (2009) and Garcia-Marques and Ferreira (2011) for recent, related arguments]. In this spirit therefore, this article and the other papers in this Research Topic examine the possible commonalities (as well as key differences) among these three key areas of cognition.

Of course, ours is not the only attempt to highlight the deeper connections between seemingly disparate cognitive activities. Logan (2002) for example, developed an instance-based theory that explains phenomena in the domains of both attention and memory. Connectionist (e.g., Kinder and Shanks, 2003; Rogers and McClelland, 2004) and Bayesian models of cognition (e.g., Kemp and Tenenbaum, 2009) often emphasize the common processing mechanisms involved in learning and memory or in different types of reasoning. To our knowledge, however, the current work is one of the first attempts to examine the links between the three domains of memory, reasoning, and categorization.

## ANALOGIES BETWEEN MEMORY AND REASONING

Elsewhere we have discussed in detail the different types of relations that may be found between two of these domains, reasoning, and memory (Heit and Hayes, 2005, 2011; Heit and Rotello, 2005; Heit et al., 2012). One notable relation is that analogous research questions have been posed in each area. For example, in both fields there is a lively and on-going debate concerning the number of core processes that drive people’s judgments. According to two-process accounts of memory (Rotello and Heit, 1999; Yonelinas, 2002; Wixted and Mickes, 2010), recognition judgments are driven by two different psychological mechanisms; a relatively fast and automatic assessment of item familiarity, and a slower, deterministic process based on item recollection. These processes are often invoked to explain functional dissociations between judgments made under instructions to recognize any item that feels familiar (“know” instructions) in contrast to recognizing items for which associated details can be retrieved (“remember” instructions). In contrast “single process” accounts (Wixted and Stretch, 2004; Dougal and Rotello, 2007; Dunn, 2008) suggest that recognition is driven by a single underlying mechanism (assessment of an item’s “memory strength”) and that the distinction between remember and know judgments can be explained by shifts in the criterion for making a response (with a more conservative criterion used for “remember” instructions; Donaldson, 1996). In terms of signal detection theory (SDT), the single-process theory explains the difference between remember and know judgments as a change in response criterion rather than memory sensitivity.

An analogous distinction between dual- and single-process models can be found in research on deductive and inductive reasoning. Descriptively, deduction involves deciding whether an inference *necessarily* follows from a given set of premises (e.g., if “Birds have property X” then it follows that “Sparrows have property X”). Induction on the other hand, involves assessing the plausibility of an inference given the premises (e.g., if “Sparrows have property X” then it seems likely that other birds share that feature, even though this is not necessarily true). Dual-process accounts (e.g., de Neys, 2006; Evans, 2008; Heit and Rotello, 2010) argue that each type of reasoning draws on qualitatively different cognitive processes. Induction is characterized as fast, intuitive, and heuristic whereas deduction is characterized as slow and making use of deterministic rules similar to those used in symbolic logic. In contrast, single-process models (e.g., Johnson-Laird, 1994; Oaksford and Chater, 2007) assume that both types of reasoning may be the result of a single common process but may differ in their decision criteria (with a more conservative criterion used in deduction; see Rips, 2001).

An important implication of this analogy is that the methods used to compare processing models in one area, such as memory, may be useful in advancing the debate in another area, such as reasoning (cf. Medin et al., 1995, for a related argument regarding parallels between issues in decision making and similarity judgment). In research on memory for example, signal detection methods have been used to evaluate single- and dual-process models of recognition. Several types of SDT patterns are seen as evidence against a single process, such as differences in sensitivity for different types of judgment, differences in the slope of receiver operating characteristic (ROC) curves, and a non-monotonic relationship between the two types of judgments across probe items (Rotello et al., 2004; Wixted and Mickes, 2010).

Heit et al. (2012) have used an analogous signal-detection approach to test single- and dual-process models of reasoning. Adapting the paradigm first used by Rips (2001), participants were asked to evaluate logically valid or invalid arguments under instructions that emphasized either deductive validity or inductive plausibility. A number of factors such as the typicality of the premise item (Heit and Rotello, 2005), the number of premises consistent with the conclusion (Rotello and Heit, 2009) and the similarity of premise and conclusion items (Heit and Rotello, 2010), were found to have a more profound impact on inductive than deductive judgments. Conversely the logical validity of arguments had more of an effect on deduction. Moreover, when people doing deduction were placed under time pressure they showed reduced sensitivity to the logical validity of items and increased sensitivity to premise-conclusion similarity; in other words, under time pressure those doing deduction made decisions that were more consistent with induction (Heit and Rotello, 2010).

**FIGURE 1:**
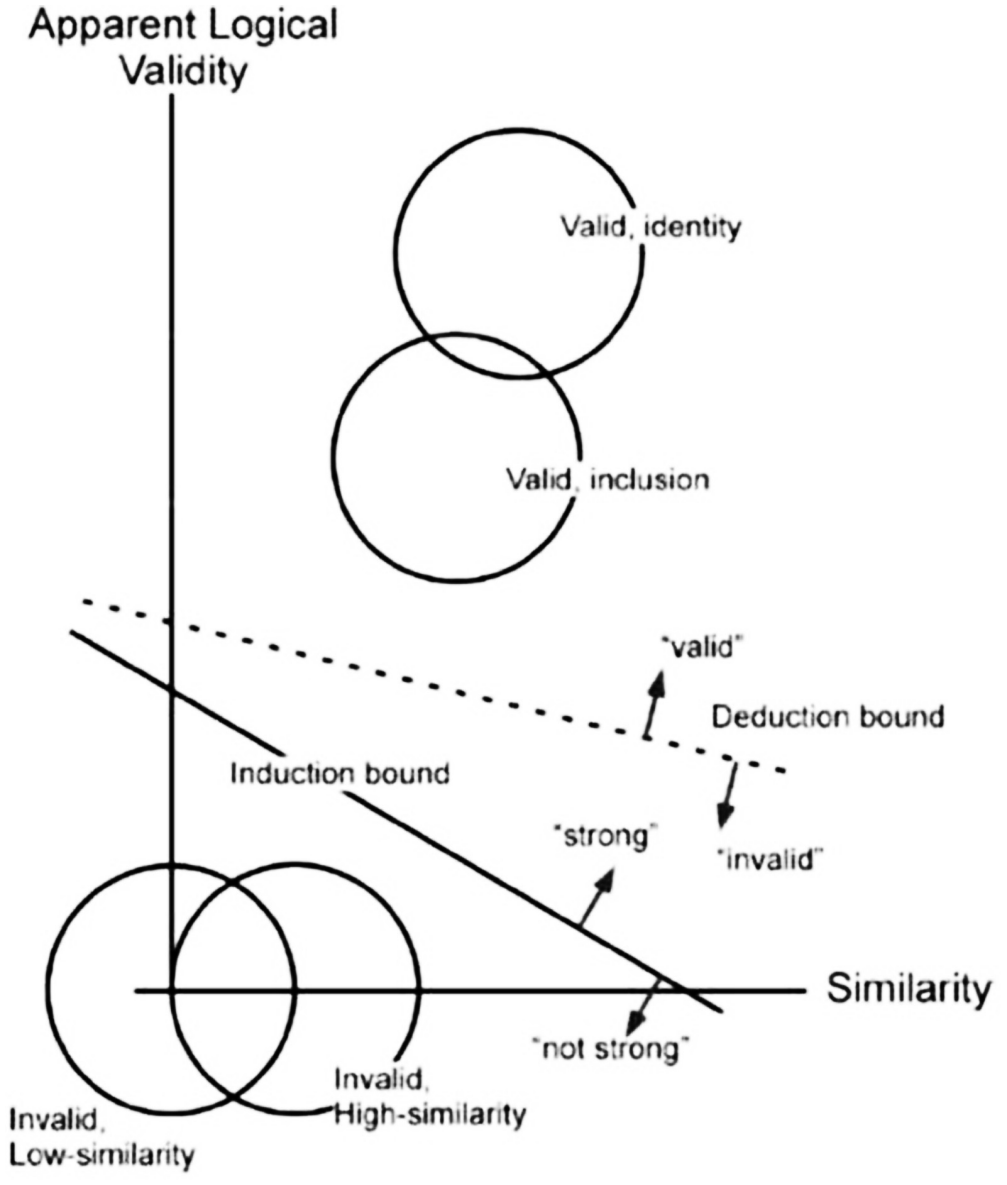
**Schematic two-dimensional signal detection model of induction and deduction.** (Reproduced from Heit and Rotello, 2010).

Signal detection analyses of these data generally favored a two-process interpretation. Induction and deduction judgments for valid and invalid items were found to differ in sensitivity, not just in response bias or criterion location (Rotello and Heit, 2009; Heit and Rotello, 2010). Confidence ratings for induction and deduction judgments were also used to construct ROC curves. Contrary to single-process predictions, the shapes of these curves also differed for induction and deduction, with significantly more area under the curve found for deduction ROCs, reflecting better discrimination between valid and invalid items. In subsequent modeling Heit and Rotello compared the fit of two signal detection models to the ROC data. The one-dimensional model assumes that the only difference between induction and deduction was in response criterion. The two-dimensional model (see **Figure 1**) assumes that two orthogonal types of information, roughly “logical correctness” and “consistency with associative knowledge,” contribute to reasoning, with different weighted proportions of each type of information used in deduction and induction. Monte Carlo simulations found that the single-process model failed to capture key trends in the data, such as differences in the sensitivity of induction and deduction judgments and the effects of argument length and item similarity. The data, however, were well explained by the two-dimensional model.

It would be premature to think that these results have settled the debate in favor of two-process models of reasoning (see Lassiter and Goodman, 2012; Stephens and Dunn, 2013 for alternative single-process interpretations of studies comparing induction and deduction). Nevertheless they can be seen as good examples of the empirical innovation and theory development that can flow from drawing analogies between issues and methods in the memory and reasoning literatures.

A further example of a positive yield from the analogy between memory and reasoning comes from applying signal detection methods to the phenomenon of belief bias in deductive reasoning. Belief bias refers to the fact that the ability to discriminate between logically valid and invalid arguments is apparently affected by the consistency of those arguments with background knowledge and beliefs (Klauer et al., 2000). Consider the following two arguments:

**Table:** 

Believable	Unbelievable
No addictive things are inexpensive	No cigarettes are inexpensive.
Some cigarettes are inexpensive	Some addictive things are inexpensive.
Therefore, some addictive things are not cigarettes	Therefore, some cigarettes are not addictive

These arguments have an identical logical structure. Neither is valid. However, because the conclusion in the first argument is consistent with people’s background beliefs it is frequently endorsed while the second argument is usually rejected (Evans et al., 1983). Likewise people often have more difficulty accepting logically valid arguments with unbelievable than believable conclusions.

The belief bias has often been measured by calculating an “interaction index.” This is the difference between two difference scores: the rate of positive responding to valid unbelievable arguments minus the response rate to invalid unbelievable arguments, and the response rate to valid believable arguments minus the response rate to invalid believable arguments. This index is usually found to be positive, reflecting the more difficult discrimination for arguments with believable conclusions.

For over three decades the interaction index has played a central role in research and theory development in the field of deductive reasoning (Klauer et al., 2000). Recent work (Dube et al., 2010, 2011; Heit and Rotello, 2014) however, suggests that this approach to measuring belief bias is flawed. This method for measuring deductive reasoning closely parallels a common approach to measuring the accuracy of recognition memory; namely the calculation of a “corrected” recognition score where the false alarm rate is subtracted from the hit rate. The validity of this measure in memory research is based on the assumption of a linear relationship between hits and false alarms. However, in memory research this assumption rarely holds (Dube and Rotello, 2012). Likewise, Heit and Rotello’s work reviewed earlier suggests that deductive judgments cannot be accounted for by a linear dimension of argument strength. In response Dube et al. (2010) collected validity judgments and confidence ratings for deductive syllogisms that varied in validity and in the believability of their conclusions. Analysis of ROC curves revealed that the belief basis effect primarily reflects the use of a more liberal response for arguments with believable as compared with unbelievable conclusions. Contrary to the assumptions of many theories of deductive reasoning, there was no evidence that sensitivity in detecting argument validity was greater for unbelievable than believable arguments. With that said, see Trippas et al. (2013) for evidence using this same methodology that there may be some exceptions based on individual differences.

## EVIDENCE FOR A DEEPER RELATIONSHIP: COMMON PROCESSES IN MEMORY AND REASONING

The work reviewed in the previous section suggests that there are strong analogies between the research questions examined in the memory and reasoning literatures. In this section we examine evidence that points to a deeper relationship between these tasks; namely that they share common processing mechanisms.

Much of this work has focused on commonalities between recognition judgments and inductive reasoning judgments (Heit and Hayes, 2011; Hayes et al., 2013; Hayes and Heit, 2013). Descriptively there seem to be good reasons for suspecting that these tasks may share common processes. Each task begins with the encoding of new information about a number of instances or exemplars. In recognition this amounts to memorizing the items presented at study. In induction this involves learning novel properties of study instances. Encoding is followed by a test phase where the goal is either to distinguish studied from new instances (recognition) or to infer which new instances have the same properties as studied items (induction). Each type of judgment is assumed to involve a comparison of the similarity of novel test probes with members of the study set (Heit and Hayes, 2011). Our thesis is that the process of similarity comparison *is the same* for recognition and induction, but that the tasks differ in how broadly responses are generalized to novel items. The specific goal of recognition decisions (is the probe similar enough to studied exemplars to conclude that it is identical?) leads to a narrower generalization of responding than in induction (is the probe item similar enough to studied exemplars to conclude that it has the same properties?).

Our strategy for testing these ideas was as follows. First we developed a research paradigm where a common study and test set was presented under either recognition or induction instructions, and we examined the empirical relationship between responses to test items in each task. Second, we manipulated a range of independent variables thought to impact the process of similarity comparison (e.g., study exposure time, exposure frequency, inclusion of child participants) and examined their effects on recognition and induction judgments. Third, we developed an exemplar-based model, GENeralization from EXamples (GEN-EX) that assumed a common processing architecture for memory and reasoning, and fitted it to our empirical data. The details of this work have been reported elsewhere (Heit and Hayes, 2011; Hayes et al., 2013; Hayes and Heit, 2013). Here, however, we illustrate this research strategy by summarizing the method and outcomes of a new study examining the relations between recognition and induction.

### ITEM CONTEXT EFFECTS IN RECOGNITION AND INDUCTION

As in previous work (e.g., Heit and Hayes, 2011; Hayes et al., 2013) this study made use of a common paradigm for studying recognition and induction. Pictures of large dogs were presented at study with instructions to memorize the items (recognition) or to learn about animals that shared a novel property (induction). At test all participants were shown the same test set, which contained studied large dogs, easy-to-reject unstudied small and medium-sized dogs, and harder-to-reject lures (new large dogs). Those in the recognition condition were instructed to respond “yes” if they thought a test item had been presented previously. Those in the induction condition were instructed to respond “yes” if they thought the test item had the same novel property as the study items.

**FIGURE 2:**
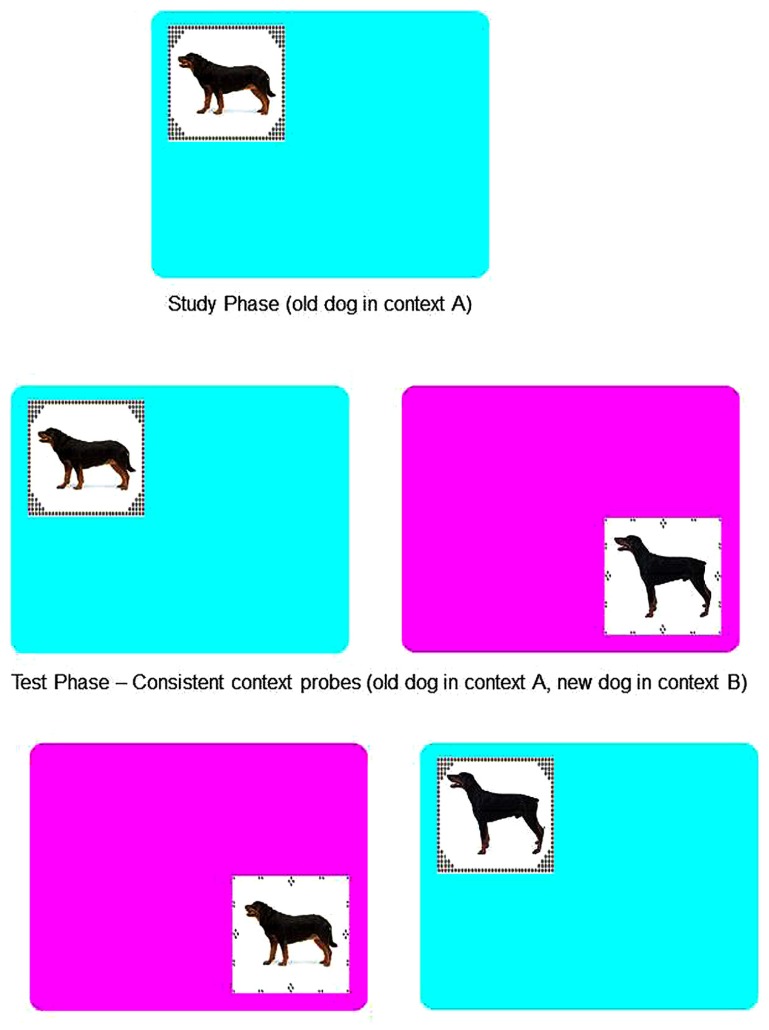
**Example of the context manipulation used in studies of recognition memory and induction**.

Notably this study also manipulated the perceptual context in which study and test items appeared. In both recognition and induction conditions pictures of dogs were presented at study in a distinctive perceptual context (i.e., surrounded by a “picture frame” on a colored background, as illustrated in the top panel of **Figure 2**). At test half of the old and new items were presented in a “consistent context,” with old items appearing in the same context as during study, and new items appearing in a novel (unstudied) context. The remaining items were presented in a “reversed context,” with old items appearing in a novel context and new items presented in one of the contexts used during the study phase (see bottom panel of **Figure 2**).

A common finding in recognition memory research is that discrimination between old and new items is affected by such contextual manipulations (see Smith and Vela, 2001 for a review). When an item is studied in a particular context and that context is reinstated at test, recognition accuracy improves. Conversely, recognition accuracy declines when an item is studied in a specific context but tested in a different context (e.g., Tulving and Thomson, 1973; Murnane and Phelps, 1993; Murnane et al., 1999). The presentation of a novel item in a familiar context can also increase false recognition of that item (e.g., Thomson, 1988), sometimes to the point that the data are better described as reflecting a criterion shift (Dougal and Rotello, 1999).

At first glance it may be hard to see why the context manipulation should affect induction. Prominent theories of induction (e.g., Osherson et al., 1990; Sloman, 1993) focus on the overlap between the taxonomic features of premise and conclusion categories as the basis of inductive projection. Such approaches make no mention of a possible role of perceptual context in property induction.

Our exemplar-based approach, on the other hand, suggests that both recognition and induction are affected by the specific similarity between familiar and novel items (Heit and Hayes, 2011). We assume that similarity computations are affected by the perceptual context in which an exemplar is embedded with matching context increasing the similarity between items and mismatching context decreasing similarity. Based on these assumptions we expected that the context changes should affect both recognition and induction. In both tasks we expected sharper discrimination between old and new test items (i.e., less positive responding to novel items) when item context remained consistent across study and test than when it was reversed.

Equal numbers of undergraduates were allocated to either a recognition or induction condition (*N* = 80). Those in the recognition condition were told to memorize study items and that their task at test was to discriminate between old and new items. Those in the induction condition were told that at study they would learn about animals that had the novel property of “beta cells” and that their task at test was to determine what other animals had this property.

At study participants in each group were shown 10 color pictures of dogs. Each picture was presented together with its context, for 2 s. After study there was a 60 s unfilled retention interval before the test phase. At test, 44 test pictures (10 old dogs, 20 new small and medium dogs, 14 “lures” or new large dogs) were presented in random order. Half were shown in a context that was consistent with that seen at study (i.e., five of the old items appeared in the same frame presented at study, and half of all the new items appeared in novel frames). For the remaining test items, context was reversed. That is, five old items appeared in novel frames and half of the new items appeared in frames that were used during study (see **Figure 2**).

**Table 1** shows the proportion of “yes” responses in each condition. The rate of positive responding to all new items was higher in induction than recognition, consistent with our expectation that participants would be more likely to generalize to novel stimuli in the induction task. For new small and medium dogs, the rate of positive responding was higher when context was reversed than when it was consistent, suggesting that the study context itself provided some degree of similarity match (e.g., Murnane and Phelps, 1994; Dougal and Rotello, 1999). Signal detection analyses found that sensitivity (*d*′) in old–new discrimination was better in recognition than in induction, *F*(1,78) = 8.83, *p* < 0.004, and better when item context remained consistent between study and test, *F*(1,78) = 24.89, *p* < 0.001. Crucially, there was no interaction between task and context (*F* < 2.5); as predicted, changing item context had analogous effects on recognition and induction.

**Table 1:** Proportion of “yes” responses and d’s for recognition and induction.

Context	Task	Old	New small and medium	Lure	*d’* (old–new)	*d’* (old–lure)
Consistent	Recognition	0.76	0.12	0.32	1.89	1.22
	Induction	0.81	0.35	0.57	1.22	0.61
Reversed	Recognition	0.70	0.17	0.30	1.18	1.11
	Induction	0.80	0.37	0.55	0.85	0.61

The other important result in this experiment relates to the relationship between the probability of making a positive response to test items in induction and recognition. The proportion of positive responses was calculated for each test item (averaged across participants) in each of the four experimental conditions, and itemwise correlations between these proportions were calculated. The correlation between recognition and induction responding was strong and positive for both context consistent, *r*(20) = 0.94, *p* < 0.001, and context reversed test items, *r*(20) = 0.95, *p* < 0.001. In other words, in both context conditions it was possible to predict the pattern of induction responses from recognition responses to the same test items, and vice versa.

We were also able to successfully model these data using a common processing architecture for recognition and induction. The GEN-EX model is embodied by two equations. Equation 1 shows the familiarity rule: The familiarity of each test stimulus equals its summed similarity to the *n* studied items, where similarity is assumed to be a negative exponential function of psychological distance between the test and study items (e.g., Nosofsky, 1986, 1988). The free parameter *c* reflects specificity of responding to test items; lower values of *c* correspond to broader generalization while higher values correspond to narrower generalization gradients.

(1) $$fam(test)=\sum_{i=1}^{n}\exp\left(-c\,\;dist\left(test,\;{study}_{i}\right)\right)$$

(2) $$resp(test)=\frac{fam\left(test\right)}{fam(test)+\beta }$$

The response rule is shown in Eq. 2. Essentially, the probability of a positive response is a monotonic function of a test item’s familiarity. The response rule has a single scaling parameter, *β*. A lower value of *β* corresponds to a greater overall tendency to respond positively. To model recognition and induction judgments using GEN-EX, we relied on empirical similarity ratings between pairs of study and test items collected in our previous work (Heit and Hayes, 2011). The GEN-EX model was able to reproduce key aspects of the data (e.g., higher response rate to new items in induction than recognition, higher response rate to new items in consistent context than reversed context), and showed good overall fit (RMSE = 0.11, correlation between model and data = 0.91). Moreover the model parameters were consistent with the theoretical predictions. When context remained consistent across study and test the c parameter was lower for induction (*c*_IND_ = 2.31) than recognition (*c*_REC_ = 3.37), indicating broader generalization of responding for induction. When context was reversed the *c* parameter was even lower for induction (*c*_IND_ = 1.89, *c*_REC_ = 3.47), reflecting a further reduction in discrimination between old and new test items (NB. the value of the *β* parameters remained relatively stable across task and context manipulations). We acknowledge that there may be other ways of modeling the effects of context on recognition and induction (e.g., similarity in context may have a multiplicative effect with the visual similarity between old and new dogs; cf. Clark and Gronlund, 1996). Nevertheless the current modeling results serve as a demonstration that the role of context in recognition and induction can be explained using the GEN-EX framework.

Both the empirical and modeling results of this study parallel those found in previous studies (Heit and Hayes, 2011; Hayes et al., 2013; Hayes and Heit, 2013). Despite a higher rate of positive responding to novel items in induction than recognition, there appear to be many striking similarities between judgments on each task. There is often a close empirical relationship between patterns of test responding to test items in induction and deduction. Moreover, manipulations like test context that affect the perceived similarity between old and new items have parallel effects on the two tasks. Finally a single exemplar-based similarity model that allows for task differences in generalization gradients can explain both recognition and induction performance.

This work strongly suggests that the links between memory and reasoning go well beyond the level of analogy. When paradigmatic differences are kept to a minimum there is good empirical and modeling evidence for shared processes between these cognitive acts. To date these links have primarily been investigated between one form of reasoning (induction) and one form of memory (recognition). There is already some evidence, however, that they might extend further. Hayes and Heit (2013) recently found evidence of a strong empirical relationship between recognition and more complex forms of induction which could involve flexible generalization along alternative property dimensions, especially when judgments were made under time pressure. Moreover a modified form of GEN-EX that allowed for the inclusion of multiple forms of perceived similarity (e.g., with respect to habitat, with respect to biology) was able to account for both recognition and these more flexible forms of induction.

## LINKING RECOGNITION AND INDUCTION TO CATEGORIZATION

In this final section we briefly consider evidence which suggests that memory and reasoning may each be linked to a third important cognitive activity, object categorization.

### INDUCTION AND CATEGORIZATION

A close relationship between category structure and induction has for some time been a key assumption of major theories of category-based induction (e.g., Osherson et al., 1990; Sloman, 1993; Medin et al., 2003). According to these approaches the structural features of natural categories profoundly affect the strength of inductive inferences. Hence inductions tend to be stronger between members of similar categories or when based on instances that are considered typical category exemplars (see Hayes et al., 2010 for a review).

There is also direct empirical evidence of the connection between categorization and inductive judgments. Sloutsky and Fisher (2004) for example, presented preschool children with animal picture triads containing a target and two probes. One probe had the same label as the target but low visual similarity whereas the other probe had a different label but high visual similarity. Different groups of children made either categorization judgments (which probe was the same kind of animal as the target?) or induction judgments (which probe shared a novel property of the target?) about a number of such triads. There was a very close correspondence between the probes that children chose in the respective categorization and induction tasks (itemwise correlation, *r* = 0.97).

A similar finding was reported by Rehder and Burnett (2005) in a study with adults using artificial categories. After learning about the typical features of a novel category and causal relations between these features, participants were presented with a feature inference task (infer the value of a missing feature given other exemplar features) followed by a categorization task (rate the likelihood that the same test item belongs to a target category). Again a strong empirical relation between induction and categorization judgments was found, especially when exemplars contained a feature that was a common cause of other features (*r* = 0.99).

### CATEGORIZATION AND RECOGNITION

Likewise, there is a body of evidence showing an empirical and theoretically meaningful relationship between categorization and recognition. Much of this evidence comes from studies of categories in which participants first learn to assign exemplars to contrasting categories and then make categorization or old–new recognition judgments about a common set of test exemplars (e.g., Omohundro, 1981; Metcalfe and Fisher, 1986; Nosofsky, 1986; Nosofsky and Zaki, 1998). These studies suggest that the empirical relationship between recognition and classification is more complicated than the relationship between recognition and induction. Often the correlation between categorization and recognition of a common test set is relatively low and there is no positive contingency between recognizing an item and categorizing it (e.g., Omohundro, 1981; Metcalfe and Fisher, 1986). Moreover, amnesics have been shown to accurately categorize tests instances that they do not recognize (e.g., Knowlton and Squire, 1993; Nosofsky and Zaki, 1998).

Crucially, however, Nosofsky (1986, 1988) and Nosofsky and Zaki (1998) have shown that such empirical dissociations between recognition and categorization are well explained by the generalized context model (GCM) which assumes a single representational system based on the storage of individual exemplars, but different decision rules in each task. Whereas recognition responses are based on the *summed* similarity of a probe to all previously experienced exemplars (as in the GEN-EX model described earlier and in the global matching models of memory; Clark and Gronlund, 1996), categorization responses are based on the *relative* similarity of the probe to the exemplars of each alternative category.

As a further test of this model Nosofsky et al. (2012) compared categorization responses to a common test set to recognition responses made under either “standard” instructions (emphasizing positive responding only on the basis of identity between old and new items) or “lax” instructions (emphasizing responding to all items that might have been old). Patterns of test responding as well as associated patterns of brain activity in the lax recognition condition were more similar to those in categorization than to standard recognition. Moreover responses on all three tasks were well described by a modified version of GCM that allowed for task differences in response thresholds.

Such demonstrations of a single processing framework underlying categorization and recognition may seem at odds with claims that category learning is driven by at least two psychologically and neurally distinct systems (e.g., Ashby and Maddox, 2011). This claim remains controversial (cf. Stanton and Nosofsky, 2007; Newell et al., 2011) and a detailed consideration lies outside the bounds of the current paper. We suggest, however, that the approach for evaluating single and dual process accounts of reasoning outlined in an earlier section might be usefully adapted to examine the issue of multiple categorization systems.

## CONCLUSION

This review has highlighted the commonalities between reasoning and memory, between categorization and reasoning, and between categorization and memory. In terms of reasoning and memory, we have shown that there is a close analogy between some of the key research questions addressed in each area. Hence, methods used to answer these questions in memory research (e.g., analysis of ROC curves) can be usefully applied to address important issues in reasoning.

The links between memory and reasoning go deeper than surface analogy, however. We have shown that performance on inductive reasoning and recognition memory tasks can be explained by a single processing model that assumes each task has a different threshold for generalizing encoded information to novel instances. This conclusion is strikingly similar to the exemplar-based account of the relationship between categorization and recognition (e.g., Nosofsky, 1988; Nosofsky et al., 2012).

**FIGURE 3:**
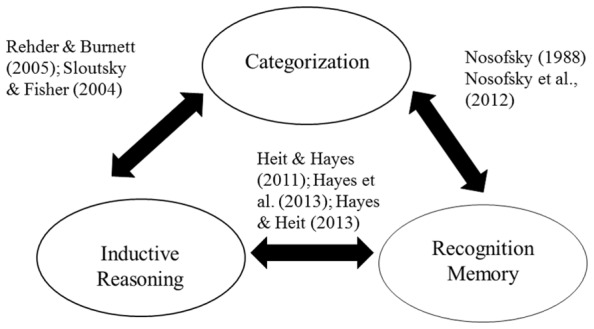
**Schematic representation of the empirical relations between recognition memory, inductive reasoning, and categorization, with example studies cited**.

We still lack direct evidence of connections among all three tasks; to our knowledge no one has yet collected inductive, recognition, and categorization judgments for the same set of stimuli. Nevertheless, the pattern of empirical links schematically summarized in **Figure 3** is highly suggestive. The Figure reinforces the view that there are processing components common to all three tasks. Our review suggests that one such component is the assessment of the total similarity between novel items and experienced items. The outputs of such similarity comparisons guide responding in each task, although the threshold for generalizing to novel items varies parametrically across the three tasks.

One implication of this review is that, as suggested by Newell (1973), we should re-think some of the conventional boundaries within the domain of cognitive science. Dividing cognition into separate domains such as memory, reasoning, categorization, and so on, may have pedagogical value (e.g., when writing textbooks or teaching classes). It may also make the study of performance in each domain more tractable, at least at an early stage of scientific progress. But it is important to keep in mind that the boundaries between cognitive activities are often due to pre-theoretical assumptions and socially constructed conventions (cf., Kuhn, 1996), rather than direct empirical comparisons or attempts to model underlying processes. One of the key aims of this review and the other invited submissions in this Research Topic was to balance the study of fractionated facets of cognition with an acknowledgment that seemingly disparate tasks may often share common component processes (see Hahn and Chater, 1998; Pothos, 2005 for related approaches). Our hope is that this will encourage other researchers to consider and to investigate the relations among different cognitive activities.

## Conflict of Interest Statement

The authors declare that the research was conducted in the absence of any commercial or financial relationships that could be construed as a potential conflict of interest.
